# A4 PREDNISOLONE, A GLUCOCORTICOID WIDELY USED FOR TREATMENT OF IBD, ENHANCES A HUMAN INTERLEUKIN-4-ACTIVATED MACROPHAGE PHENOTYPE

**DOI:** 10.1093/jcag/gwac036.004

**Published:** 2023-03-07

**Authors:** R S Deshpande, B E Callejas Pina, R Peng, J A Sousa, A Wang, R Panaccione, D M McKay

**Affiliations:** 1 Gastrointestinal Research Group and Inflammation Research Network, Department of Physiology & Pharmacology, Snyder Institute for Chronic Diseases, Cumming School of Medicine; 2 Division of Gastroenterology and Hepatology, Department of Medicine, University of Calgary, Calgary, Canada

## Abstract

**Background:**

With cellular immunotherapy, the individuals’ medication could ablate (or enhance) any therapeutic benefit of the transferred cells. Murine and human macrophages activated with IL-4 (i.e., M(IL4)) improve wound healing and reduce the severity of disease in murine models of colitis. Advancing the position that autologous M(IL4) could be a novel approach to IBD, a critical question arises: will concurrent medication impact the M(IL4)s anti-colitic effect? To address this, we tested if prednisolone, a synthetic, anti-inflammatory glucocorticoid used to induce remission in IBD flares,impacts human M(IL4) phenotype and function.

**Purpose:**

To determine if prednisolone suppresses or enhances a human M(IL4) phenotype as defined by canonical marker molecules and wound healing and anti-colitic activities.

**Method:**

Macrophages were differentiated from the blood monocytes of healthy volunteers using M-CSF (7 days) and treated with GMP-grade IL-4 (10 ng/mL, 48h) ± a 24h treatment with prednisolone (1μg/mL). Subsequently, conditioned medium was collected for TGFb measurement by ELISA and for use in a T84 epithelial cell *in vitro* wound healing assay. Retrieved M(IL4) and M(IL4,pred.) were characterized by mRNA expression of CD206 (mannose receptor), RAMP1 (CGRP receptor), and CD14 (LPS co-receptor). One million murine bone marrow-derived M(IL4) or M(IL4,pred.) were injected into BALB/c mice 48h prior to intra-rectal DNBS (3mg), and colitis was assessed 72h-post DNBS.

**Result(s):**

Human M(IL4)s displayed increased mRNA expression of CD206 and RAMP1, and reduced CD14 compared to M(0), with the CD206 and RAMP1 being further increased by prednisolone treatment. M(IL4,pred.) produced more TGF-β than M(IL4) upon LPS stimulation [363 ± 30 vs. 241 ± 24 pg/ml, n= 4, p<0.05], which would predict an enhanced wound healing capacity. Stimulated M(IL4,pred.) produced more IL-10 than M(IL4). Furthermore, murine M(IL4,pred.) retained an anti-colitic capacity comparable to M(IL4) as determined by disease activity score in the DNBS model.

**Conclusion(s):**

Human M(IL4)s subsequently exposed to the potent immunomodulatory glucocorticoid, prednisolone show increased expression of phenotypic markers and increased output of TGFb and IL-10. Crucially M(IL4,pred.) retained an anti-colic effect in the murine DNBS model of colitis. Interpreting these data, we suggest that the anti-colitic effect of M(IL4) immunotherapy would not be adversely offset by the individuals concomitant use of steroids. Our preliminary findings support pursuing M(IL4) transfers as a novel approach to the management of IBD.

**Please acknowledge all funding agencies by checking the applicable boxes below:**

Other

**Please indicate your source of funding;:**

Helmsley Charitable Trust

**Disclosure of Interest:**

None Declared

